# Bidirectional associations between brain functional networks and sleep disorders: A bidirectional Mendelian randomization study

**DOI:** 10.1097/MD.0000000000050778

**Published:** 2026-09-18

**Authors:** Lixia Dong, Hanliang Wei, Tingting Wu, Junxing Dong, Tengteng Fan, Weifeng Mi, Sanwang Wang

**Affiliations:** a Department of Neurology, Zhengzhou Seventh People’s Hospital, Zhengzhou, Henan, China; b Institute of Brain Science and Brain-inspired Research, Shandong First Medical University & Shandong Academy of Medical Sciences, Jinan, Shandong, China; c Peking University Sixth Hospital, Peking University Institute of Mental Health, NHC Key Laboratory of Mental Health (Peking University), National Clinical Research Center for Mental Disorders (Peking University Sixth Hospital), Beijing, China; d Institute of Basic Medical Sciences, Chinese Academy of Medical Sciences & Peking Union Medical College, Beijing, China.

**Keywords:** bidirectional association, brain functional network, Mendelian randomization, rsfMRI, sleep disorder

## Abstract

The prevalence of sleep disorders has been rising over recent decades. Sleep disorders are frequently associated with disruptions in resting-state functional brain networks. Despite these associations, the direction and genetic basis of the relationships between brain functional networks and sleep disorders remain unclear. Utilizing bidirectional two-sample Mendelian randomization (MR), we examined bidirectional associations between 191 resting-state functional magnetic resonance imaging (rsfMRI) phenotypes (n = 34,691 individuals) and 8 sleep disorder phenotypes (N = 307,843–410,385 individuals). Forward MR identified 14 rsfMRI traits that showed significant associations with sleep disorder phenotypes. Notably, the inverse variance-weighted method showed that increased amplitude in the precuneus or occipital regions within the default mode or central executive network was associated with 13.2% higher odds of sleep disorders (combined; odds ratio = 1.132, 95% confidence interval: 1.064–1.204, *P* = 8.30 × 10^−5^). In addition, reverse MR identified 6 sleep disorder phenotypes with significant associations involving 18 rsfMRI traits. For example, higher genetic liability to sleep disorders (combined) was associated with a 14.2% lower strength of functional connectivity between precentral, frontal, or supplementary motor and frontal networks (odds ratio = 0.858, 95% confidence interval: 0.795–0.927, *P* = 9.62 × 10^−5^). This study identified 32 bidirectional MR associations between sleep disorders and brain functional networks, providing candidate neural circuits for future mechanistic and interventional studies of sleep disorders.

## 1. Introduction

Sleep is an essential physiological state for sustaining human life.^[1]^ Accumulating epidemiological evidence indicates that sleep disorders represent a major public health burden; for example, a recent systematic review estimated that insomnia affects approximately 16.2% of the global adult population, corresponding to more than 850 million adults worldwide.^[2]^ Clinically, sleep disorders encompass several partially overlapping categories, including insomnia, sleep-related breathing disorders, circadian rhythm sleep-wake disorders, sleep-related movement disorders, central disorders of hypersomnolence, parasomnias, and other or unspecified sleep disorders.^[3]^ In the present study, we focused on 8 registry-based sleep disorder phenotypes available from FinnGen, including sleep disorders (combined), insomnia, sleep apnea-related phenotypes, disorders of the sleep-wake schedule, and other or unspecified sleep disorders. These sleep disorders are further related to a variety of adverse health outcomes,^[4]^ including cardiovascular disease,^[5]^ Alzheimer disease,^[6]^ diabetes,^[7]^ and psychiatric disorders.^[8]^ Therefore, there is an urgent requirement for effective interventions and treatments for sleep disorders to ensure effective management. However, our current understanding of the pathophysiology of sleep disorders remains limited, impeding efficient interventions and treatments.

Studies have described the anatomical abnormalities of the brain in sleep disorders,^[9–13]^ suggesting that cortical structural dysfunction may be implicated in such conditions. In recent years, Mendelian randomization (MR) studies have reported genetically informed associations between structural abnormalities in brain regions and sleep disorders.^[14–16]^ For instance, insufficient sleep has been reported to increase the surface area of the lateral occipital cortex and decrease the thickness of the paracentral cortex,^[16]^ whereas insomnia has been associated with increased surface area of the medial orbitofrontal cortex.^[14]^ Correspondingly, the likelihood of insomnia decreases with greater global cortical surface area.^[14]^ Nevertheless, the relationship between brain anatomy and function is nonlinear,^[17]^ and structural abnormalities do not necessarily translate into functional abnormalities. Indeed, several brain regions often operate in concert to support specific brain processes. Therefore, it is important to examine the bidirectional association between sleep disorders and functional brain networks. In particular, interconnections and communication among brain networks may contribute to the regulation of cognition, behavior, and emotion. Resting-state functional magnetic resonance imaging (rsfMRI) is an effective noninvasive method for measuring communication between brain regions. Functional MRI uses blood oxygen level-dependent signals to measure brain activity, based on the differential magnetic properties of deoxyhemoglobin and oxyhemoglobin.^[18]^ Coordinated interactions among brain regions during coactivation are commonly conceptualized as network systems. Key brain networks involved in cognitive processes include the default mode network (DMN), salience network (SN), and central executive network (CEN).^[19]^ A growing body of research indicates that sleep disorders are associated with dysfunction in brain functional networks. For instance, patients with chronic insomnia showed higher functional connectivity (FC) between the anterior cingulate cortex (ACC) and the right middle frontal gyrus and lower connectivity between the ACC and the bilateral precuneus compared with healthy controls.^[20]^ Reduced FC in the posterior and anterior SN, bilateral executive control network, and ventral DMN has also been observed in patients with obstructive sleep apnea (OSA).^[21]^ Abnormal cerebrocerebellar resting-state FC^[22]^ and hypoconnectivity within the hippocampal and caudate functional networks^[23]^ have also been reported in OSA. In patients with idiopathic hypersomnia, resting-state FC was reduced within the anterior DMN (medial prefrontal cortex) and was associated with self-reported daytime sleepiness.^[24]^ In addition, drug-naive patients with idiopathic restless legs syndrome showed reduced FC in the cuneus, fusiform gyrus, paracentral lobule, and precuneus.^[25]^ Evidence further suggests that different chronotypes show distinct FC responses to homeostatic sleep pressure: as homeostatic sleep pressure increased, negative FC between the right insula and bilateral angular gyri increased in morningness-chronotype participants but decreased in eveningness-chronotype participants.^[26]^ Together, these findings indicate disruptions of brain FC in sleep disorders. Nevertheless, the precise nature of the association between abnormalities in functional brain networks and sleep disorders remains unclear. Exploring the bidirectional association between brain functional phenotypes and sleep disorders is essential for understanding the pathophysiology of sleep disorders and identifying candidate biological pathways.

Recent large-scale genetic studies have made it possible to explore the bidirectional genetically informed associations between sleep disturbances and brain functional networks. MR analysis facilitates causal inference by leveraging genetic evidence from rsfMRI and sleep disorder studies.^[17,27,28]^ Because alleles are randomly assorted during gametogenesis, MR can provide evidence about the direction and magnitude of genetically proxied associations when its assumptions are satisfied. In MR, genetic variants associated with an exposure serve as instrumental variables (IVs) for estimating the association between the exposure and the outcome.^[29]^

In this work, we examined the relationships between 8 traits of sleep disorders and 191 brain rsfMRI traits using bidirectional two-sample MR analyses, and 32 possible causal relationships were identified, including 14 resting-state brain functional traits that causally influence the risk of sleep disorders and 18 resting-state brain functional traits that are causally affected by sleep disorders. In addition to offering support for potential causal relationships between brain functional network alterations and sleep disorders, our findings provide candidate networks that may be further evaluated in mechanistic, longitudinal, and interventional studies.

## 2. Methods

### 2.1. GWAS data sources

We obtained rsfMRI genome-wide association study (GWAS) summary statistics from a study of 34,691 UK Biobank (UKB) participants, in which 9,026,427 common genetic variants were evaluated in relation to 1777 intrinsic brain activity phenotypes.^[30]^ Of the 1777 intrinsic brain activity phenotypes, 191 showed genome‑wide significant genetic associations (*P* < 5 × 10^−8^) in the source GWAS and thus yielded potentially suitable genetic instruments for MR. These phenotypes comprised 5 global FC measures, 111 pairwise FC measures (edges), and 75 amplitude measures (nodes) reflecting localized spontaneous brain activity. In the subsequent MR analysis, a phenotype was retained only when at least 4 independent instrumental single-nucleotide polymorphisms (SNPs) remained after linkage disequilibrium (LD) clumping, confounder screening, outcome-association screening, and exposure–outcome harmonization.

To minimize potential confounding effects arising from participant overlap between GWASs of sleep disturbances and fMRI traits in the MR analysis, we incorporated GWAS findings on sleep disorders that excluded UKB samples. In addition, to reduce the risk of spurious MR estimates attributable to genetic heterogeneity and other contextual variables, we restricted our analysis to GWAS datasets comprising individuals of European ancestry. According to these criteria, the GWAS data for sleep disorders were obtained from the latest release of the FinnGen project. Sleep disorder phenotypes in FinnGen were registry-based endpoints defined primarily using International Classification of Diseases codes from nationwide health registries rather than self-reported symptoms. The analysis covered 8 sleep disorder phenotypes, including a combined sleep disorder category and more specific categories related to insomnia, sleep apnea, circadian sleep-wake schedule disorders, and other or unspecified sleep disorders. One endpoint was labeled by FinnGen as “sleep apnoea, including avohilmo.” AvoHILMO is the Finnish national Register of Primary Health Care Visits, which captures diagnoses recorded in outpatient primary healthcare. Accordingly, this endpoint refers to sleep apnea with additional case ascertainment through the AvoHILMO register; it does not denote a separate sleep apnea subtype or a comorbid disorder. The diagnostic definitions, International Classification of Diseases code mappings, numbers of cases and controls, and ancestry information for each phenotype are summarized in Table 1. All disease classifications adhere to the International Classification of Diseases, 10th Revision standards.

**Table 1:** Data source characteristics for the Mendelian randomization study.

Trait	Data sources	Sample size	Ancestry
Insomnia	FinnGen	4214 cases and 371,145 controls	European
Sleep disorders, other/unspecified	FinnGen	893 cases and 405,229 controls	European
Disorder of the sleep-wake schedule	FinnGen	456 cases and 405,229 controls	European
Sleep apnoea	FinnGen	43,901 cases and 366,484 controls	European
Sleep disorders (combined)	FinnGen	49,880 cases and 358,194 controls	European
Other sleep disorders	FinnGen	3588 cases and 312,803 controls	European
Nonorganic sleeping disorders (more control exclusions)	FinnGen	5964 cases and 301,879 controls	European
Sleep apnoea, including avohilmo	FinnGen	46,975 cases and 365,206 controls	European

This study was based exclusively on publicly available GWAS summary statistics and did not involve individual-level participant data. Ethical approval and informed consent had been obtained in the original contributing studies, including UKB and FinnGen. Therefore, additional institutional review board approval was not required for the present secondary analysis.

### 2.2. Instruments selection

Three core assumptions underpin MR analysis: the IVs should be strongly associated with the exposure; the IVs should be independent of confounders; and the IVs should influence the outcome only through the exposure of interest.^[31]^ To meet these assumptions, we prescreened the instruments before conducting the MR analysis. First, we selected SNPs that were strongly associated (*P* < 5 × 10^−8^) with the exposure. Subsequently, we excluded SNPs associated with confounding variables that could disrupt the relationship between brain rsfMRI traits and sleep disorders. Based on an extensive literature review, smoking, BMI, and depression were identified as factors that may affect both sleep disorders^[32–34]^ and brain FC.^[35–37]^ Consequently, SNPs significantly associated with these phenotypes were excluded: for instance, rs12721051, rs1113879, rs6772953 (associated with smoking); rs9783783, rs12721051 (associated with BMI); and rs2678894, rs2717067, and rs2867610 (associated with depression). Detailed information on the remaining confounding factors is provided in Supplementary eTable 1, Supplemental Digital Content 1. The LDlink website was used to validate SNPs and mitigate the potential confounding effects of variants that might be associated with sleep disorders.^[38]^ Third, we eliminated the SNPs related to the outcome (*P* < 5 × 10^−8^) and the SNPs in high LD with variants linked to the outcome using the clump function in the R package TwoSampleMR (MRC Integrative Epidemiology Unit, University of Bristol).^[39]^ All the IVs we used can be obtained in Supplementary eTable 2, Supplemental Digital Content 2.

### 2.3. Bidirectional two-sample MR analyses

The relationships between sleep disturbances and rsfMRI traits were assessed through bidirectional two-sample MR analyses.^[40]^ In the forward MR analyses, brain rsfMRI traits were used as exposures and sleep disorder phenotypes were used as outcomes; in the reverse MR analyses, sleep disorder phenotypes were used as exposures and rsfMRI traits were used as outcomes. In the forward MR analyses, effect estimates for binary sleep disorder outcomes were reported as conventional odds ratios (ORs). In the reverse MR analyses, the underlying estimates for continuous rsfMRI outcomes were beta coefficients. Following the reporting framework of the previously published bidirectional MR study,^[17]^ these coefficients and their confidence limits were exponentiated and reported as ORs for consistency of presentation across analytical directions (OR = exp [β]). In the reverse analyses, OR denotes an exponentiated beta estimate rather than a conventional OR for a binary rsfMRI outcome. An OR >1 indicates a positive association, and an OR <1 indicates an inverse association. *P* values were derived from the original untransformed beta coefficients and standard errors. The random-effects inverse variance-weighted (IVW) method was utilized as the primary method because of its precise estimates by combining SNP-exposure-to-SNP-outcome ratios in a meta-analysis. However, it assumes no directional horizontal pleiotropy, constraining the regression intercept to zero.^[41]^ Therefore, MR-Egger, weighted-median, weighted mode, and simple mode were applied to enhance the robustness of the MR estimates. The MR-Egger method assumes that instrument strength is independent of the direct effect and produces MR effect estimates consistent with the slope coefficient in Egger regression.^[42]^ The weighted-median approach calculates MR estimates by employing the median of the weighted empirical density function of individual SNP effect estimates, allowing for up to 50% of genetic variants to exhibit horizontal pleiotropy.^[43]^ The weighted mode assumes that the majority of variants are valid, clustering SNPs based on similarity of MR estimates and estimating the MR effect from the cluster with the most instruments. It yields consistent estimates when multiple SNPs are valid.^[44]^ To ensure the robustness of MR findings, stringent criteria were applied to both forward and reverse MR analyses: a minimum inclusion of 4 SNPs as IVs, consistent directional alignment across all estimated MR effects, and attainment of a Bonferroni-corrected *P*-value threshold of <1.31 × 10^−4^ (0.05/191/2). Adherence to these criteria was required to identify a robust MR association between the exposure and outcome variables.

### 2.4. Sensitivity analyses

Sensitivity analyses were performed to detect horizontal pleiotropy that could violate the MR assumptions. MR-Egger intercept tests, Cochran’s *Q* test, leave-one-out analyses, the Mendelian Randomization Pleiotropy RESidual Sum and Outlier (MR-PRESSO) global test, and the MR Steiger test were conducted to assess the reliability of the results. Horizontal pleiotropy was assessed using MR-Egger regression^[45]^ and MR-PRESSO global tests.^[46]^ The Cochran *Q* test was conducted for the IVW model to evaluate heterogeneity. Leave-one-out analysis was performed to determine whether an MR estimate was driven by a particular SNP. To remove any possible perturbation from overlapping IVs across exposure phenotypes, we reanalyzed MR after excluding these overlapping SNPs.^[41]^ The MR Steiger directionality test was used to investigate the inferred direction from exposure to outcome. In addition, the 8 sleep disorder endpoints were treated as distinct clinical hypothesis families because they differed in diagnostic definition, ascertainment, and clinical interpretation and were not statistically interchangeable. As a sensitivity analysis, we also considered the more conservative global threshold of 3.37 × 10^−5^ (0.05/191/8) within each direction. Findings with *P* values between the global and phenotype-specific thresholds should be interpreted cautiously.

### 2.5. Genetic correlation analysis

Linkage disequilibrium score regression is an advanced analytical technique based on GWAS summary-level data. It is used to evaluate the heritability of complex traits and the genetic correlation between different traits. The core idea is to use LD to estimate the contribution of each SNP to overall genetic variation. Whole-genome genetic correlation (rg) refers to the degree of shared genetic variation between 2 traits, quantifying the average genetic effect shared between traits while reducing the influence of environmental confounding. The rg value ranges from −1 to 1, indicating a negative to positive genetic correlation. In the presence of a genetic correlation between an exposure and the outcome of interest, the interpretation of MR estimates may be complicated.^[47,48]^

## 3. Results

### 3.1. Summary of the research

**Figure 1.**
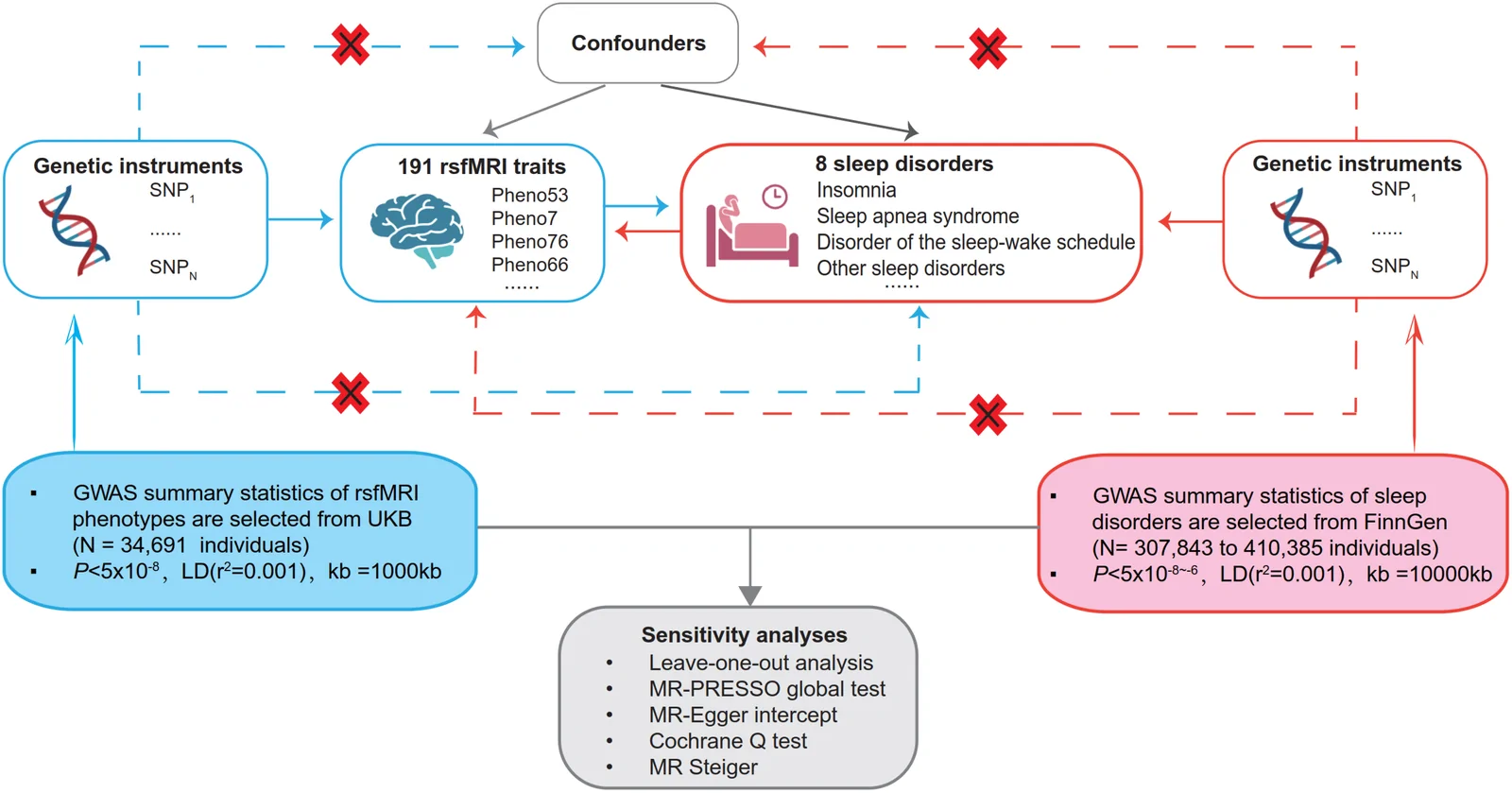
Flowchart of this bidirectional two-sample Mendelian randomization analysis. The analysis included 2 directions: forward MR, in which 191 rsfMRI traits were used as exposures and 8 sleep disorder phenotypes were used as outcomes, and reverse MR, in which sleep disorder phenotypes were used as exposures and rsfMRI traits were used as outcomes. Genetic instruments were selected based on genome-wide significance, linkage disequilibrium clumping, and exclusion of variants associated with potential confounders. Sensitivity analyses included leave-one-out analysis, the MR-PRESSO global test, the MR-Egger intercept test, Cochran’s *Q* test, and the MR Steiger directionality test. GWAS = genome-wide association study, LD = linkage disequilibrium, MR = Mendelian randomization, MR-PRESSO = Mendelian Randomization Pleiotropy RESidual Sum and Outlier, rsfMRI = resting-state functional magnetic resonance imaging, SNP = single-nucleotide polymorphism, UKB = UK Biobank.

To investigate possible links between brain resting-state functional networks and sleep disorders, we used GWAS summary statistics of rsfMRI phenotypes from Zhao et al^[30]^ and 8 sleep disorder phenotypes to conduct bidirectional two-sample MR analyses. Because genetic effects on brain function are relatively modest compared with those on structural attributes,^[49]^ only the 191 traits significantly influenced by genetic variation were selected for GWAS analysis. These traits included 5 global FC measures, 111 pairwise FC measures (edges), and 75 amplitude traits (nodes). Figure 1 provides the study flowchart. To adhere to the MR assumptions, we selected only SNPs that were significantly associated with the exposure (*P* < 5 × 10^−8^) as IVs. In addition, the robustness of the IVs was determined by computing the *F*-statistics, and all *F*-statistics of IVs were >10, which validated the strength of the IVs (Methods). Furthermore, serial sensitivity analyses were performed to evaluate the robustness in both forward and reverse MR inferences. The evidence previously presented indicates that the IV selection was reasonable and supports the robustness of the bidirectional MR estimates.

### 3.2. Forward MR analysis of rsfMRI traits and sleep disorders

**Figure 2.**
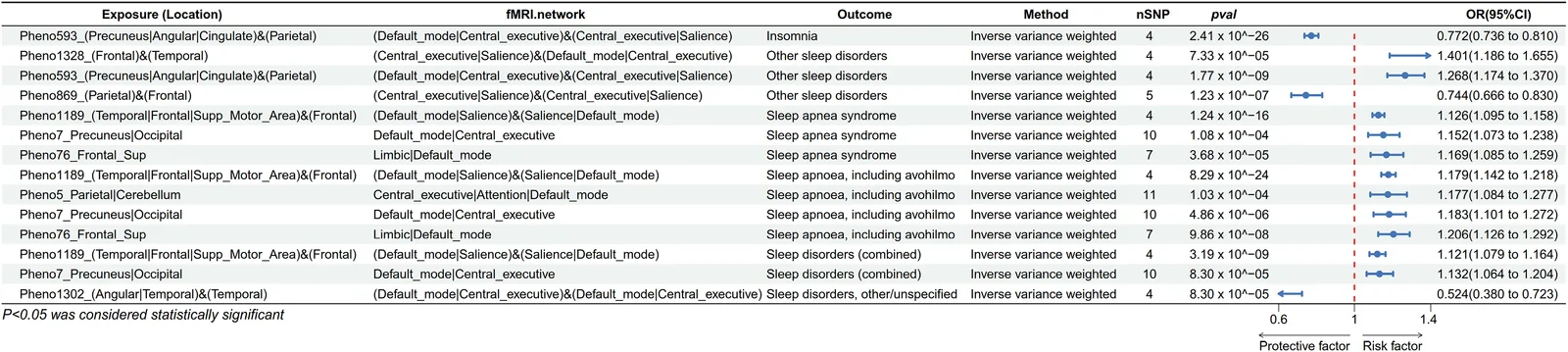
Forest plot of MR estimates and their 95% CIs for associations of rsfMRI traits with sleep disorders. Each row represents one significant forward MR association, with an rsfMRI phenotype as the exposure and a sleep disorder phenotype as the outcome. The fMRI.network column indicates the large-scale functional network or network pair involved in each rsfMRI phenotype. ORs >1 indicate that higher genetically predicted rsfMRI activity or connectivity is associated with increased liability to the corresponding sleep disorder, whereas ORs <1 indicate a protective association. Estimates were obtained using the inverse variance-weighted method. The vertical dashed line indicates OR = 1. *P* values were interpreted using the Bonferroni-corrected threshold, and *P* < 1.31 × 10^−4^ was considered statistically significant. CI = confidence interval, MR = Mendelian randomization, nSNP = number of single-nucleotide polymorphisms, OR = odds ratio, rsfMRI = resting-state functional magnetic resonance imaging.

Fourteen rsfMRI phenotypes showed significant associations with sleep disorder phenotypes in the forward MR analysis. As shown in Figure 2, except for insomnia and other/unspecified sleep disorders, a one-standard-deviation increase in the amplitude trait or the FC of different brain regions in the DMN was associated with a 12% to 40% higher risk of sleep disorders. The OR (95% confidence interval [CI]) for neuronal activity in the frontal, temporal, or supplementary motor and frontal lobes during a rest state on “sleep disorders (combined)” was 1.121, as calculated by the IVW method ([1.079–1.164], *P* = 3.19 × 10^−9^), indicating that a one-standard-deviation increase in the FC of the default mode or SNs raises the risk of “sleep disorders (combined)” by 12.1%. In addition, the OR (95% CI) for neuronal activity in the precuneus or occipital region on “sleep disorders (combined)” was 1.132, as calculated by the IVW method ([1.064–1.204], *P* = 8.30 × 10^−5^), indicating that each standard-deviation increase in functional activity in the default mode or CEN was associated with a 13.2% higher risk of “sleep disorders (combined).” All detailed forward MR results are shown in Supplementary eTable 3, Supplemental Digital Content 3.

#### 3.2.1. rsfMRI traits affect the risk of insomnia

Insomnia is one of the most common sleep disorders characterized by difficulty falling asleep, difficulty maintaining sleep, and early awakening.^[4]^ By the IVW approach, our study found that increased FC between the angular/precuneus region and parietal region (within the DMN, CEN, or SN) reduced the risk of insomnia by 22.8% (OR = 0.772 [0.736–0.810], *P* = 2.41 × 10^−26^; Fig. 2).

#### 3.2.2. rsfMRI traits affect the risk of sleep apnea

Sleep apnea syndrome (SAS) is a sleep disorder in which breathing is frequently interrupted. The main types of SAS are OSA and central sleep apnea, which can lead to physiological and psychological consequences, including hypertension, right heart failure, and death.^[50]^ In our study, we identified 4 rsfMRI traits that showed significant forward MR associations with sleep apnea. These traits were related to activity in the temporal or frontal or supplementary motor and frontal regions; the precuneus or occipital region; the superior frontal region; and the parietal or cerebellar regions. They mainly involved the DMN, SN, CEN, limbic network, and attention network (Fig. 2). The 3 primary networks (DMN, SN, and CEN) facilitate effective cognitive processing. Mounting evidence suggests that disruption of these networks contributes to many mental and sleep disorders.^[17,21,51]^ The results of our study demonstrated that the OR calculated by the IVW method (95% CIs) for neural activity in the frontal or temporal or supplementary motor and frontal lobe during rest on SAS and “sleep apnoea, including avohilmo” were 1.126 ([1.095–1.158], *P* = 1.24 × 10^−16^) and 1.179 ([1.142–1.218], *P* = 8.29 × 10^−24^), respectively. Therefore, each standard-deviation increase in the FC of the default mode or SNs increased the likelihood of suffering sleep apnea by 12.6% and “sleep apnoea, including avohilmo” by 17.9%. In the default mode or the CEN, the increased functional amplitude of one standard deviation in the precuneus or occipital region was linked to a 15.2% higher risk of SAS (OR = 1.152 [1.073–1.238], *P* = 1.08 × 10^−4^) and an 18.3% higher risk of “sleep apnoea, including avohilmo” (OR = 1.183 [1.101–1.272], *P* = 4.86 × 10^−6^). The amplitude feature measures the degree of neuronal activity in the area of interest by reflecting changes in the rsfMRI signal.^[52]^ In the limbic or the DMN, a rise of one standard deviation in the functional amplitude of the superior frontal area was correlated with a 16.9% higher likelihood of suffering from SAS (OR = 1.169 [1.085–1.259], *P* = 3.68 × 10^−5^) and a 20.6% higher risk of “sleep apnoea, including avohilmo” (OR = 1.206 ([1.126–1.292], *P* = 9.86 × 10^−8^)). Notably, the attention network was specifically linked to the likelihood of suffering from “sleep apnoea, including avohilmo”; a one-standard-deviation increase in the functional amplitude of the parietal or cerebellar area was linked to a 17.7% higher risk of “sleep apnoea, including avohilmo” (OR = 1.177 [1.084–1.277], *P* = 1.03 × 10^−4^).

#### 3.2.3. rsfMRI traits affect the risk of other or unspecified sleep disorders

We found that 4 rsfMRI features showed significant forward MR associations with other and unspecified sleep disorders (Fig. 2). Two rsfMRI features were negatively associated with other/unspecified and other sleep disorders, as calculated by the IVW method, including angular or temporal and temporal gyrus (OR = 0.524 [0.380–0.723], *P* = 8.30 × 10^−5^) and parietal or frontal gyrus (OR = 0.744 [0.666–0.830], *P* = 1.23 × 10^−7^). Two rsfMRI phenotypes in the default mode, salience, and CENs were positively correlated with the other sleep disorders. Specifically, a one-standard-deviation increase in the FC of the frontal or temporal region was linked to a 40.1% higher risk of other sleep disorders (OR = 1.401 [1.186–1.655], *P* = 7.33 × 10^−5^), and the incidence of other sleep disorders increased by 26.8% (OR = 1.268 [1.174–1.370], *P* = 1.77 × 10^−9^) for every standard-deviation increase in the FC of the precuneus, angular, cingulate, and parietal areas.

### 3.3. Reverse MR analysis of sleep disorders and rsfMRI traits

**Figure 3.**
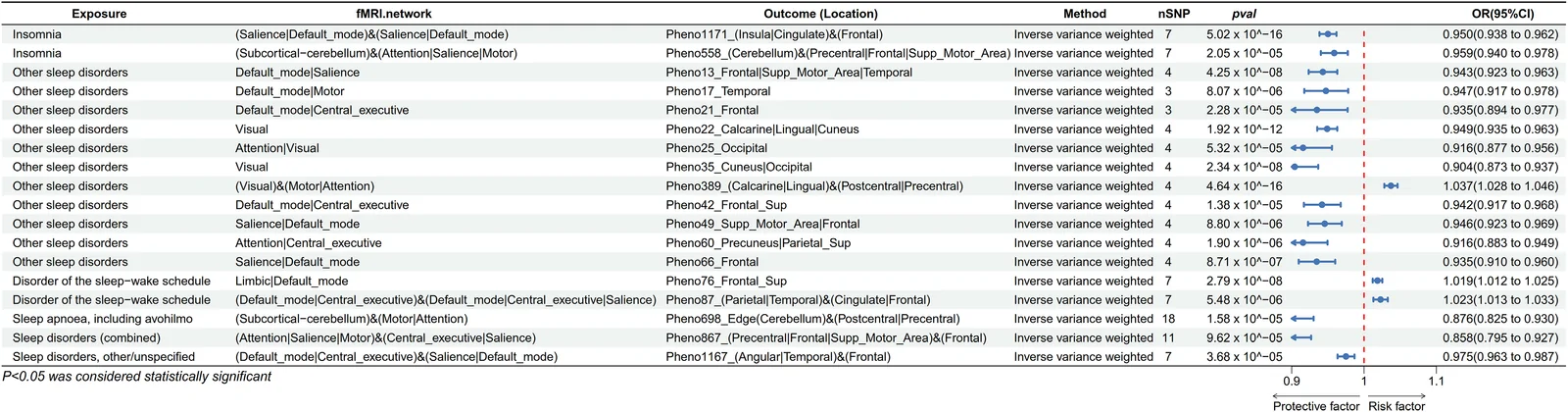
Forest plot of MR estimates and their 95% CIs for the effect of sleep disorders on rsfMRI traits. Each row represents one significant reverse MR association, with a sleep disorder phenotype as the exposure and an rsfMRI phenotype as the outcome. The fMRI.network column indicates the large-scale functional network or network pair corresponding to the affected rsfMRI phenotype. ORs >1 indicate that higher genetically predicted liability to a sleep disorder is associated with increased rsfMRI activity or connectivity, whereas ORs <1 indicate reduced rsfMRI activity or connectivity. Estimates were obtained using the inverse variance-weighted method. The vertical dashed line indicates OR = 1. *P* values were interpreted using the Bonferroni-corrected threshold, and *P* < 1.31 × 10^−4^ was considered statistically significant. CI = confidence interval, MR = Mendelian randomization, nSNP = number of single-nucleotide polymorphisms, OR = odds ratio, rsfMRI = resting-state functional magnetic resonance imaging.

We also evaluated reverse associations between genetic liability to sleep disorders and rsfMRI phenotypes. Eighteen significant reverse MR associations were identified (Fig. 3). Apart from “sleep apnoea, including avohilmo” and “sleep disorders (combined),” genetic liability to sleep disorders was mainly associated with spontaneous neural activity or FC in brain regions within the DMN. Higher genetic liability to sleep disorders was generally associated with lower functional measures in the DMN. In particular, weakened connectivity in the precentral, frontal, supplementary motor, and frontal regions may result from “sleep disorders (combined)” (OR = 0.858 [0.795–0.927], *P* = 9.62 × 10^−5^). Detailed reverse MR results can be found in Supplementary eTable 4, Supplemental Digital Content 4.

### 3.4. Insomnia affects the functional networks in the brain

Two significant reverse MR associations were observed between the risk of insomnia and rsfMRI traits in the reverse MR analysis (Fig. 3). The main findings calculated by the IVW method showed that the increased risk of insomnia reduced the FC of the insula or cingulate and frontal region in the DMN and SN (OR = 0.950 [0.938–0.962], *P* = 5.02 × 10^−16^). In addition, insomnia may reduce the strength of FC in the cerebellum and precentral or frontal or supplementary motor areas (OR = 0.959 [0.940–0.978], *P* = 2.05 × 10^−5^).

#### 3.4.1. Disorder of the sleep-wake schedule affects the functional networks in the brain

We identified two significant reverse MR associations between genetic liability to disorder of the sleep-wake schedule and rsfMRI phenotypes in the reverse MR estimates (Fig. 3). Estimated by the IVW method, a higher risk of disorder of the sleep-wake schedule was positively linked to the functional activity of the superior frontal area (OR = 1.019 [1.012–1.025], *P* = 2.79 × 10^−8^) and the parietal or temporal and cingulate or frontal networks (OR = 1.023 [1.013–1.033], *P* = 5.48 × 10^−6^).

#### 3.4.2. SAS affects the functional networks in the brain

As indicated in Figure 3, genetically predicted risk of “sleep apnoea, including avohilmo” was associated with reduced FC between the cerebellum and postcentral/precentral regions within the subcortical–cerebellar and motor/attention networks (OR = 0.876 [0.825–0.930], *P* = 1.58 × 10^−5^).

#### 3.4.3. Other and unspecified sleep disorders affect the functional networks in the brain

We revealed 12 associations between other or unspecified sleep disorders and rsfMRI traits in the reverse MR analysis (Fig. 3). Apart from the aforementioned DMN, the risk of other and unspecified sleep disorders was also negatively associated with the amplitude traits or connectivity of 11 rsfMRI phenotypes in central executive, salience, motor, attention, and visual networks. However, the risk of other sleep disorders was positively correlated with the connectivity of the calcarine or lingual and postcentral or precentral regions in the visual and motor or attention networks (OR = 1.037 [1.028–1.046], *P* = 4.64 × 10^−16^).

#### 3.4.4. Sensitivity analyses

Consistent tendencies are shown in the scatter plots (Supplementary Figs. 1 and 2, Supplemental Digital Content 5) for both the forward and reverse analyses. By either the MR-PRESSO global method or the MR-Egger intercept test, no horizontal pleiotropy was found by MR-Egger (Supplementary eTables 1, 2, and 5, Supplemental Digital Content 1) or MR-PRESSO (sensitivity analysis, *P* > .05; Supplementary eTables 1 and 2, Supplemental Digital Content 1). The MR Steiger analysis supported the inferred direction from brain functional network traits to sleep disorder phenotypes (Supplementary eTables 1, 2, and 6, Supplemental Digital Content 1). Cochran’s *Q* test (*P* > .05) revealed no significant heterogeneity (Supplementary eTables 1, 2, and 7, Supplemental Digital Content 1). Moreover, the leave-one-out plots (Supplementary Figs. 3 and 4, Supplemental Digital Content 10) showed that the results were not significantly affected by the removal of an SNP, supporting the stability of the MR associations.

### 3.5. Genetic correlation analysis

In this study, we used linkage disequilibrium score regression to examine genetic correlations between 191 rsfMRI phenotypes and 8 sleep disorder phenotypes. As shown in Supplementary eTable 8, Supplemental Digital Content 12, a total of 1528 results were obtained across all sleep disorder phenotypes, of which 124 showed significant correlations (*P*_rg_ < .05) with rsfMRI, including 2 MR analyses. The identified candidate phenotypes, Pheno66 and Pheno593, showed genetic correlation with other sleep disorders. No additional evidence supported significant genetic correlations between other rsfMRI and sleep disorder phenotypes. This finding is consistent with previous reports and further confirms the robustness of our results.

## 4. Discussion

We examined the relationships between 8 types of sleep disorders and 191 brain rsfMRI traits using bidirectional, two-sample MR analyses. Our findings provide evidence for associations between sleep disorders and resting-state functional brain networks. Specifically, the present study revealed effect estimates for brain functional networks on sleep disorders, as well as the possibility that brain functional networks may influence the risk of sleep disorders. To the best of our knowledge, this is the first study to systematically characterize bidirectional MR associations between sleep disorders and brain functional networks, and our findings provide insight into the pathophysiology of sleep disorders. The clinical relevance of these findings is underscored by the high population burden and frequent comorbidity of sleep disorders. Recent global evidence estimated that insomnia affects approximately 16.2% of adults worldwide.^[2]^ In addition, insomnia and OSA frequently co-occur: a systematic review and meta-analysis reported that 38% of patients with OSA had insomnia and that 35% of patients with insomnia met criteria for OSA based on an apnea-hypopnea index threshold of at least 5 events per hour.^[53]^ These data highlight the value of examining shared and disorder-specific neural pathways. To provide a more coherent mechanistic framework, our findings can be interpreted along 3 interrelated dimensions. First, alterations involving the DMN may reflect dysregulation of internally oriented cognition, self-referential processing, and the transition between quiet wakefulness and sleep. Second, alterations involving the SN and CEN may reflect impaired switching between internal and external information processing and altered arousal regulation. Third, cerebellar and motor-related network findings may be particularly relevant to sleep-related breathing and sensorimotor components of sleep disorders. Because MR estimates are based on genetic instruments and summary-level data, these interpretations should be regarded as biologically plausible hypotheses rather than direct demonstrations of neural mechanisms.

Notably, we identified MR associations between resting-state neural activity or FC in the DMN and sleep disorders. Strengthened connectivity within functional areas of the DMN increased the likelihood of sleep disorders, suggesting that DMN hyperactivation may be 1 pathophysiological pathway underlying sleep disorders. The DMN comprises widely distributed brain regions in the parietal, temporal, and frontal cortex that are involved in internally oriented attention. These regions decrease their neural activity during complex attention-demanding tasks (e.g., social cognition and semantic and episodic memory) and increase their activity during rest or quiet wakefulness.^[54]^ Converging studies have demonstrated a crucial role of the DMN in sleep-wake regulation,^[55]^ and functional abnormalities in the DMN have been widely reported in sleep disorders.^[22,56,57]^ A prior study showed a correlation between decreased sleep quality and hyperconnectivity between the DMN and the ventral attention network, which processes salience.^[58]^ Additionally, in the current study, we also found hyperconnectivity between the DMN and the network involved in driving cognitive tasks. Therefore, hyperactivation of the default network may lead to disturbances in the balance between internal and external information processing, which in turn leads to abnormal sleep.^[19]^ In sleep apnea, abnormal network connectivity in the DMN has been reported; increased FC between the prefrontal and temporal regions was associated with reduced verbal fluency in OSA^[22,57]^ and DMN connectivity was increased in sensorimotor-associated circuits in patients with restless legs syndrome.^[59]^ According to a recent study, patients with generalized anxiety disorder and insomnia responded better to low-frequency repetitive transcranial magnetic stimulation of the right dorsolateral prefrontal cortex, and the percentage change in sleep from pre-to-post was substantially correlated with a decrease in within-DMN FC,^[60]^ suggesting that modulation of DMN-related functional alterations may be associated with changes in sleep symptoms. However, these findings provide only indirect evidence and do not establish that DMN-related genetic liability can be directly translated into therapeutic targets. Consistent with a previous study, our MR results also showed that higher FC in the precuneus, angular, cingulate, and parietal regions within the DMN and other core networks was associated with lower liability to insomnia.^[17]^ It is possible that differences in FC within the DMN, as well as heterogeneity in FC between the DMN and other networks,^[54]^ contributed to the discrepant estimated effects of the DMN on different sleep disorders. Further prospective studies are needed to clarify the directionality and biological significance of the association between the DMN and different sleep disorders.

Consistent with previous findings of reduced DMN FC in sleep deprivation^[61]^ and chronic insomnia,^[62]^ our reverse MR study also revealed that the amplitude or connectivity of the DMN was generally inversely associated with increased susceptibility to sleep disturbances. These results suggest that enhanced FC of the default network may contribute to the onset of sleep disorders and that, once sleep disorders occur, they may lead to deficits in the default network and compromise neural activity. However, for disorders of the sleep-wake schedule, we observed that a higher risk of this phenotype increased amplitude traits and FC in the DMN. Considering that hyperactivity of the DMN was positively associated with the risk of sleep disorders, we infer that disorders of the sleep-wake schedule may be linked to the pathology of other sleep disorders.^[63]^ At the same time, hyperactivity of FC in the DMN is also associated with a higher risk of depression.^[17]^ Our results suggest that disorders of the sleep-wake schedule may contribute to depression through abnormal function of the DMN, expanding the biological mechanisms by which circadian disruption contributes to the onset of depression.^[64]^ Interventions targeting circadian abnormalities and related brain networks may therefore have potential for depression prevention.

In addition to the DMN, our work also identified MR associations between other networks and sleep disorders. The insula, together with the ACC, prefrontal cortex, and periaqueductal gray, constitutes a core structure of the SN.^[65]^ The SN recognizes and modulates significant, remarkable, or uncommon information in internal and external contexts.^[66]^ We discovered that increased activity in the SN was linked to a higher likelihood of sleep disorders, such as sleep apnea and “sleep disorder (combined).” In particular, disturbance of the SN may impair muscle responsiveness and the arousal threshold to negative pharyngeal pressure and hypoxia and contribute to the development of SAS.^[67]^ The forward MR investigation also found a significant correlation between the likelihood of sleep apnea and neural connectivity activity in the superior frontal gyrus (limbic network). The development of post-traumatic stress disorder (PTSD) has been suggested to be linked to the superior frontal gyrus, which is thought to regulate amygdala activity and modulate stress perception.^[17,68]^ Notably, there is evidence connecting PTSD to an increased risk of OSA.^[69]^ These findings suggest that alterations in stress perception caused by superior frontal gyrus dysfunction may be one of the common pathogenetic mechanisms of OSA and PTSD. In addition, for disorders of the sleep-wake schedule, we observed that a higher risk of this phenotype increased amplitude traits of the superior frontal gyrus in the limbic network. Previous studies have demonstrated that disruption of sleep-wake rhythmicity is associated with a higher risk of major depressive disorder and other mood disorders,^[70,71]^ suggesting that the superior frontal gyrus in the limbic network, which is involved in emotion regulation, may be a crucial hub linking circadian disruption and mood disorders.

Our study also identified bidirectional associations between abnormal FC in the cerebellar region and sleep apnea. The cerebellum is traditionally believed to coordinate motor demands,^[72]^ but it also has connectivity with numerous brain regions, including cortical areas and limbic systems. Moreover, studies have shown that the cerebellum is involved in respiratory modulation.^[73]^ Therefore, we speculated that abnormalities in cerebellar network function may contribute to impaired coordination of respiration during sleep, thereby increasing the risk of sleep apnea. The cerebellum also plays roles in motor learning, emotion, and other cognitive functions.^[74]^ Consequently, aberrant behavior, psychological symptoms, and cognitive performance in patients with sleep apnea may be directly related to decreased FC in the cerebellar network following the onset of sleep apnea. Similarly, we found that an increased risk of insomnia reduced FC in the cerebellar region, a result that appears to contrast with a previous case-control study.^[75]^ In that study, increased FC in the cerebellum was observed in patients with chronic insomnia and was interpreted as a consequence of compensatory enhancement of language and emotion processing. Because the direction and mechanism of this relationship are not established by the present data, longitudinal studies are needed to characterize the association between functional changes in the cerebellum and insomnia.

In this study, our primary multiple-testing procedure treated each sleep disorder endpoint as a distinct prespecified hypothesis family and corrected for 191 rsfMRI phenotypes and 2 analytical directions within each endpoint. A global correction across all 8 sleep disorder endpoints would produce a more stringent threshold of *P* < 3.27 × 10^−5^. Most of the strongest associations remained significant under this global threshold, whereas several associations with *P* values between 3.27 × 10^−5^ and 1.31 × 10^−4^ should be interpreted more cautiously. Because some of the FinnGen sleep disorder endpoints are clinically related or partially overlapping, the global Bonferroni procedure may be conservative. Nevertheless, replication in independent datasets is required, particularly for findings that did not survive the global threshold.

A strength of this study is the use of genetic instruments in MR, which can reduce susceptibility to some forms of confounding and reverse causation compared with conventional observational analyses when the MR assumptions are satisfied. An additional strength is the use of rsfMRI data, which can detect more subtle variations in the function of specific brain regions and provide evidence for links between abnormalities in brain function and sleep disorders.^[76]^

Additionally, there are certain limitations to this study. First, given that the GWAS datasets utilized represent European populations, further investigation is warranted to corroborate our findings across diverse demographic groups. Second, the sleep disorder phenotypes were heterogeneous and registry-based. For example, sleep apnea includes central sleep apnea and OSA, which have different pathogenetic mechanisms, and the combined sleep disorder category aggregates conditions with distinct clinical features. This heterogeneity may introduce variability into MR estimates and limit the specificity of interpretations for any single sleep disorder subtype. Third, although we used multiple sensitivity analyses to assess pleiotropy and robustness, residual horizontal pleiotropy or phenotype misclassification cannot be fully excluded. Fourth, while our present MR analysis has uncovered certain potential associations linking abnormal network connectivity with sleep disorders, these findings predominantly rely on statistical inference and necessitate subsequent mechanistic, experimental, and clinical longitudinal assessment for validation. Notwithstanding the aforementioned limitations, this study provides an initial genetic epidemiological framework for understanding associations between rsfMRI characteristics and sleep disorders.

## 5. Conclusion

In conclusion, this bidirectional MR study identified 32 significant associations between 191 rsfMRI traits and 8 sleep disorder phenotypes. The findings suggest that abnormalities in large-scale functional brain networks, particularly the DMN, SN, CEN, and cerebellar-related networks, may contribute to the genetic liability to sleep disorders or may be influenced by sleep disorder liability. These findings provide genetic evidence linking brain functional network organization with sleep disorder risk and highlight candidate neural circuits that warrant further investigation in longitudinal and mechanistic studies.

## Acknowledgments

We would like to acknowledge the participants and investigators of the FinnGen study. We are grateful to the UK Biobank consortium investigators and the research team that provided the data for this study, as well as to all the data platform developers.

## Author contributions

**Conceptualization:** Tengteng Fan, Weifeng Mi, Sanwang Wang.

**Formal analysis:** Lixia Dong, Hanliang Wei, Tingting Wu.

**Project administration:** Tengteng Fan, Weifeng Mi, Sanwang Wang.

**Supervision:** Tengteng Fan, Weifeng Mi, Sanwang Wang.

**Writing – original draft:** Lixia Dong, Hanliang Wei, Tingting Wu.

**Writing – review & editing:** Lixia Dong, Hanliang Wei, Tingting Wu, Junxing Dong, Tengteng Fan, Weifeng Mi, Sanwang Wang.
























